# Nematicidal Activity of Fosthiazate Against Soybean Cyst Nematode *Heterodera glycines*

**DOI:** 10.21307/jofnem-2019-021

**Published:** 2019-04-23

**Authors:** Hai Yan Wu, Man Luo, Lu Yuan Zhang, Xun Bo Zhou

**Affiliations:** 1 Guangxi Key Laboratory of Agric-Environment and Agric-Products Safety, Agricultural College of Guangxi University, Nanning, 530004, China

**Keywords:** Fosthiazate, *Heterodera glycines*, Nematicidal activity, Inhibition hatching, Mortality

## Abstract

Nematicidal activity at different concentrations of fosthiazate against soybean cyst nematode (*Heterodera glycines*) was evaluated in this paper. The mortality rates of second-stage juvenile (J2) reached 13.43, 48.39, 66.82, 79.77, and 86.35% at 12 hr after exposure to 2.18, 3.44, 5.45, 8.61, and 13.62 mg/l of fosthiazate, respectively, whereas cumulative hatching rates totaled 58.24, 53.88, 42.54, 24.11, and 13.69% at 18 days after exposure to concentrations. J2s dead by exposure to fosthiazate exhibited shrunk and twisted body shape, whose length of nematode body, stylet, and esophageal glands to head were significantly shorter than that of the control (*p* < 0.05). A pot test was also performed to count the numbers of cysts on soybean roots, showing reduction of 43.64–97.94% due to application of fosthiazate at 5.45, 13.62, 34.04, and 85.10 mg/l concentrations. This study demonstrated that fosthiazate exhibits increasing of J2 mortality, and reducing egg hatching and reproduction rates, which providing evidence to support the use fosthiazate in further studies against *H. glycines*.

Soybean (*Glycine max,* Family Leguminosae) is widely grown worldwide due to its unique property of possessing high plant-based protein contents, lipid minerals, and vitamins (Olaoye and Ade-Omowaye, 2011). Soybean cyst nematode (SCN, *Heterodera glycines*) infection is the most serious disease affecting soybean production worldwide; this infection was reported for the first time in 1899 in Northeast China, since then, this nematode has been spread widely in various regions, including Asia, America, and Europe, and has become an important factor limiting soybean production globally (Wrather et al., 2001). The cysts of SCN can generally survive in the soil for 3–4 years, and eggs of *H. glycines* cyst may remain viable for up to 11 years (Inagaki and Tsutsumi, 1971). As a soil borne pathogen, SCN can be harmful for soybean during the whole growth period; the infected soybean plants show stunting and yellowing aboveground (Niblack et al., 2006). SCN caused approximately 90 billion tons of losses for the top 10 soybean-producing countries (USA, China, Brazil, Argentina, Indonesia, Canada, India, Paraguay, Italy, and Bolivia) in 1998, and the total yield losses caused by SCN in these countries were higher than those caused by any other disease (Wrather et al., 2001). The soybean seed yield (bushels) suppression by SCN in 2009 in 28 US states approximated 3.3 billion kg (Koenning and Wrather, 2010).

Numerous chemical methods have been used to control SCN; nematicide is an important management tool, but effective control of SCN has been a challenging problem (Raddy et al., 2013). Increasing concerns to human and environment safety led to the widespread deregistration of several agronomic important nematicides; thus, new and safer nematicides are urgently needed to date (Lai et al., 2014). Fosthiazate, an organophosphorus (OP) nematicide developed by Ishihara Sangyo Kaisha, Ltd., was registered and marketed in Japan in 1992; it is widely used for controlling *Meloidogyne incognita*, *H. glycines, pratylenchus penetrans*, as well as characterized by a marked systemic action against various species of insects and mites on the foliar part (Koyanagi et al., 1998). In the USA, more than 50 OP/Carbamates pesticides were registered in 2000, accounting for more than almost half of all pesticide sales (Gray and Hammitt, 2000). Fosthiazate was registered in 2004 and has been identified as a viable alternative to the use of methyl bromide for the control of nematodes infesting tomato fields (US Environmental Protection Agency, 2004). Currently, the USA is also committed to reducing the risk of pesticides and working on alternatives to OP (US Environmental Protection Agency, 2018).

Fosthiazate can act on the nervous system of targeted nematode pest, inhibit acetylcholinesterase (AChE), and block normal nerve impulse conduction (Xu, 2007). The efficacy of fosthiazate in controlling pests on tomato (*M. incognita*), potato (*Agriotes* spp., *Globodera pallida*, and *G. rostochiensis*), peanuts (*M. arenaria* and *Frankliniella* spp.), banana (*Cosmopolites sordidus*, *Meloidogyne* spp., *Hoplolaimus seinhorsti*, *Helicotylenchus multicinctus*, and *Radopholus similis*), and tobacco (*M. javanica*, *M. arenaria*, and *M. incognita*) has been evaluated in various studies and consistently confirmed its high efficiency as well as its key role in pest control (Minton et al., 1993; Rich et al., 1994; Grove et al., 2000; Chabrier et al., 2002; Saad et al., 2011).

Root-knot nematode is the most frequently target nematode in nematicidal activity determination of fosthiazate; however, limited relevant research focused on the effect of fosthiazate on cyst nematodes; only potato cyst nematode (*G. pallida* and *G. rostochiensis*), cereal cyst nematode (*H. avenae*), and tobacco cyst nematode (*Globodera tabacum solanacearum*) had been studied (Johnson, 1995; Tobin et al., 2008; Saad et al., 2011; Zhang et al., 2014).

This study aimed to investigate the following: nematicidal activity of fosthiazate on second-stage juvenile (J2) *H. glycines*; hatching-inhibition effectiveness on free eggs in vitro; effect of fosthiazate on reproduction of SCN using a pot test. The results provide important information on fosthiazate for further studies controlling *H. glycines* and field precision applications.

## Materials and methods

### Chemicals

Fosthiazate (85.1%) was obtained from the Zhengbang Bio-chemical Co., Ltd. (Nanchang, China). In bioassay, five fosthiazate concentrations (2.18, 3.44, 5.45, 8.61, and 13.62 mg/l) were used in experiments. The used concentrations were based on the relationship of fosthiazate and nematode mortality assessed in a previous experiment. When the five concentrations were increased by 1.58 times, nematode mortality ranged from 10 to 90% at 12 hr after exposure in the experiment. Concentrations of 2.18, 5.45, 13.62, 34.04, and 85.10 mg/l were used in the pot test according to lethal concentrations (LC90) at 12 hr in bioassay. Dimethyl sulfoxide (DMSO, Tianjin, China) was used as a dissolvent. The controls included 0.5% DMSO and distilled water in all experiments.

### Nematode culture and fresh J2 collection

Pot with 200 ml autoclaved fine sand (diameter 850 µm), and 50 cysts were inoculated, and susceptible soybean (cultivar, Ludou 4) was sowed in pots, with three seeds each pot, and grown in intelligent illumination incubator SPX–250B–G (Shanghai Boxun Industry and Commerce Co., Ltd, Shanghai, China) at 25°C with a 14 hr/10 hr (light/dark) photoperiod and 50–75% relative humidity. The cysts were harvested from 41-day cultures and extracted with sieving-decanting method, as described by Liu (1995). All cysts were transferred into a 50 µm pore diameter hatching sieve, surface-sterilized using 70% ethanol for 3 min, and then washed four times with distilled water. Subsequently, the hatching sieve with cysts was placed in a petri dish added with 2 ml distilled water and incubated at 25°C in darkness. Fresh infective J2 were collected from the bottom of petri dishes on the day of the experiment.

### Effects of fosthiazate concentrations on SCN J2

For each fosthiazate concentration, a total of 200 ml were dispensed into a 96-well plate with 40–50 nematodes in each well (four replicates). The process was repeated three times for each concentration, and the assay plates were kept at 25°C. Nematodes in plates were observed at 0, 2, 4, 6, 8, 10, and 12 hr after exposure under an inverted compound microscope XD30A (Sunny Optical Technology (Group) Co., Ltd, Yuyao, China) and the immobile nematodes were recorded.

At each observation time point, knockdown rates of J2 were calculated: Knockdown rate (%) = (number of knockdown J2)/(total number of J2) × 100%. In this study, the knockdown J2 indicates the ceased motor behavior of nematodes after treatment with different concentrations of fosthiazate. Then, mortality rate of J2 was calculated using the equation: mortality rate (%) = (number of dead J2)/(total number of J2) × 100%. In the present study, mortality rate of J2 in each concentrations at different exposure times were evaluated. In order to judge the nematode was really dead or fake dead, when the respective fosthiazate concentration was replaced with distilled water, the touch test occurred after 12 hr, J2s were considered really dead if nematodes could not recover upon immersion in water and showed no movement when touched with a hair needle.

### Effects of fosthiazate on the body lengths of J2

During the study, 13.62 mg/l of fosthiazate showed significant effect on *H. glycines* J2 morphology. To describe the morphological changes, fosthiazate was replaced with distilled water at 12 hr after exposure of J2 to 13.62 mg/l fosthiazate and to confirm whether the nematodes can be recovered with distilled water. Temporary nematode slides were prepared at 60 hr after recovery with water, and digital images were obtained under an inverted compound microscope ECLIPSE Ti–S (Nikon Corporation, Tokyo, Japan). The length of stylet, hyaline region of the tail, entire body, and the distance from esophageal glands to the head were measured using the software NIS–Elements D 4.30.00. The J2s treated for 72 hr with distilled water were used as control.

### Effects of fosthiazate concentrations on SCN hatching of free eggs

Cysts were collected from the roots 41 days after inoculation. Fresh eggs were released and treated with different concentrations of fosthiazate as described above. Distilled water and 0.5% DMSO solutions were used as control. The eggs were dispensed in a 96-well plate and subjected to five concentrations of fosthiazate, with four replicates, and the process was repeated three times. The assay plates were incubated at 25°C and kept in the dark. The J2 that hatched from free eggs were recorded under an inverted compound microscope at 0, 3, 6, 9, 12, 15, and 18 days after exposure to different concentrations of fosthiazate. The accumulative hatching rate was calculated using the following formula:

Accumulativehatchingrate(%)=(hatchedJ2)/(totalnumberofeggs)×100%.

### Pot test

Nematode reproduction on soybean in different treatments was investigated in pot experiments using a completely randomized design with two replicates. Seeds were placed in a petri dish with a filter paper after rinsing for five times in sterile water and germinated at 25°C. The germinated seeds with the same shoot length were retrieved after 2 days. One germinated seed was sowed in a pot (7 × 7 × 8 cm) with autoclaved fine sand. Seedling plants were inoculated with 3,000 eggs per plant at 3 days after transplanting by injection. Three holes (1 cm depth) and about 1 cm from the main stem of the plant were made in each pot. The egg suspension was transferred using a pipet which was subsequently covered with surface sand. Soybean seedlings were grown at 25°C under a 14 hr light and 10 hr dark photoperiod in an intelligent illumination incubator, and the moisture was kept constant. A total of 2 ml of each fosthiazate concentration was applied to each pot at 4 days after sowing, and water and 0.5% DMSO used as control with four replicates. Nematode reproduction was checked at 35 days after inoculation. The whole roots and all sand in the pot were collected, the root system was washed with tap water, and cysts on the roots and sand were extracted by sieving-decanting method. The cysts were collected on a 177 µm mesh sieve and hand-picked with a dissecting needle under a stereomicroscope. All cysts on the roots and sand were counted, eggs were released from the cysts and average eggs per cyst were calculated. In this study, there was no significant difference in the average eggs per cyst among different treatments. Nematode reproduction was expressed as the total number of cysts per one pot with soybean plant. The decline rate of cysts was calculated as follows:

Declinerateofcysts(%)=(cystnumberofcontrol−cystnumberoftreatment)/cystnumberofcontrol×100%.

### Statistical analysis

Statistical analysis was performed with SPSS 12 (SPSS Inc., Chicago, IL). Least significant difference tests were performed, and differences with *p* < 0.05 were considered statistically significant. All graphs were drawn by SigmaPlot 10.0.

## Results

### Nematicidal activity of fosthiazate

**Figure 1:**
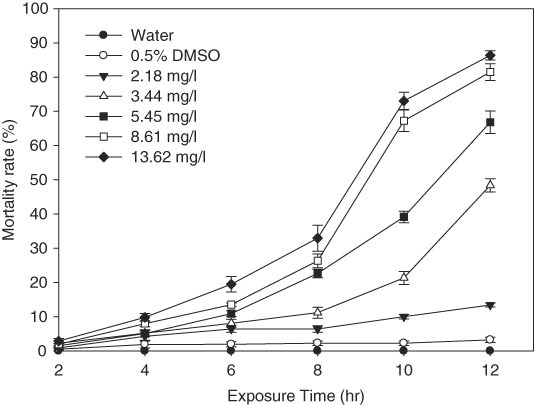
Mortality rate (%) of *H. glycines* J2 exposure to fosthiazate with exposure time.

Fosthiazate exhibited a strong toxic activity against *H. glycines* J2 at 5.45~13.62 mg/l the concentration. Overall, mortality of nematodes increased with the duration of exposure in different concentrations of fosthiazate (Table 1). No nematodes were recovered after fosthiazate was replaced with distilled water for 12 hr at corresponding exposure time, that is, the mortality rate of J2 was equal to the knockdown rate. A significant difference in mortality was observed among treatments and control at 12 hr after exposure (*p* < 0.05). The mortality rate of J2 in 2.18, 3.44, 5.45, 8.61, and 13.62 mg/l fosthiazate reached 13.43, 48.39, 66.82, 79.77, and 86.35%, respectively. At 6 hr after exposure, mortality of J2 in different concentration was significantly higher than that of control of DMSO and water (*p* < 0.05), and mortality at 13.62 mg/l concentration was significantly higher than that of other treatments (*p* < 0.05), was 19.48%. At 10 and 12 hr after exposure, nematode mortality each concentration was significantly higher than that of controls (*p* < 0.05), mortality of J2 in 13.62 mg/l concentration was 73.04 and 86.35%, respectively. At the same concentration, nematode mortality increased with increased duration of exposure (Fig. 1). In the concentration of 5.45 mg/l, there was no significant difference in mortality between 2 and 4 hr exposure, but they were significantly lower than other exposure time (*p* < 0.05). In 8.61 and 13.62 mg/l concentration, there were significant differences in different treatment time (*p* < 0.05).

**Table 1:** Mortality rate (%) of *H. glycines* J2 at different durations after exposure to different concentrations of fosthiazate.

	Exposure time (hr)					
Concentration (mg/l)	2	4	6	8	10	12
13.62	2.86 ± 0.74 a	9.80 ± 1.15 a	19.48 ± 2.29 a	32.96 ± 3.81 a	73.04 ± 2.52 a	86.35 ± 1.33 a
8.61	1.97 ± 0.86 ab	7.96 ± 0.85 a	13.54 ± 0.48 b	26.32 ± 2.04 b	67.23 ± 3.14 b	79.77 ± 2.72 b
5.45	1.55 ± 0.61 abc	5.07 ± 0.90 b	10.90 ± 0.86 bc	22.64 ± 1.22 b	39.15 ± 1.68 c	66.82 ± 3.26 c
3.44	2.24 ± 0.70 ab	5.17 ± 0.99 b	8.10 ± 1.16 cd	11.18 ± 1.62 c	21.35 ± 1.88 d	48.39 ± 1.93 d
2.18	0.89 ± 0.60 bc	4.29 ± 1.20 bc	6.41 ± 0.98 d	6.41 ± 0.98 cd	10.01 ± 0.65 e	13.43 ± 0.57 e
0.5% DMSO	0.52 ± 0.52 bc	1.93 ± 1.00 cd	1.90 ± 0.73 e	2.27 ± 0.68 de	2.27 ± 0.68 f	3.23 ± 0.80 f
Water	0.00 ± 0.00 c	0.00 ± 0.00 d	0.00 ± 0.00 e	0.00 ± 0.00 e	0.00 ± 0.00 f	0.00 ± 0.00 f

**Notes:** Numbers followed “±” referred to standard error. Means within the same column followed by different letters are significantly different (*p* < 0.05) according to LSD test.

Nematicidal activity of fosthiazate was evaluated by comparing the median lethal concentrations (LC50) for different concentrations on *H. glycines* under different exposure times. LC50 were 3912.59, 25.00, and 4.41 mg/l fosthiazate at 4, 8, and 12 hr exposure, respectively. The LC50 and LC90 values decreased with prolonged exposure time (Table 2).

**Table 2:** Toxicity of fosthiazate to *H. glycines* J2 at different treatment exposure time.

Exposure time (h)	Slope(±SE)	Correlation coefficient	LC50 (95%CI)	LC90 (95%CI)
4	0.54 (± 0.10)	0.95	3912.59 (317.10−48275.85)	941773.86 (9,541.28−92957,985.01)
8	1.38 (± 0.18)	0.98	25.00 (16.53−37.80)	212.43 (83.90−537.87)
12	2.65 (± 0.44)	0.97	4.41 (3.53−5.50)	13.41 (9.36−19.20 )

**Note:** LC, lethal concentration expressed in mg/l fosthiazate with 95% confidence intervals (CI).

### Effects of fosthiazate on the morphology of J2

**Figure 2:**
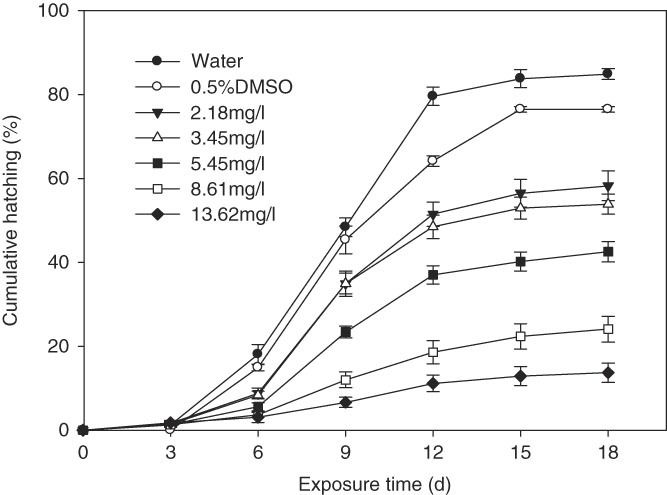
The shape of *H. glycines* J2. (A, B) normal shape of J2; (B) natural shape of normal dead J2; (C, D) the shape of dead J2 killed by fosthiazate. Bar: A, C:100 µm, B, D:50 µm.

The shape of J2 killed by fosthiazate (curved; Figs 2C 2D) differed from the straight appearance of natural-death J2 (Fig. 2A 2B). Fosthiazate can shorten the body length of J2s. The dead nematodes cannot move with a twisted body shape and shrunk. The length of nematode body, stylet, and the distance from the esophageal glands to head were significantly shorter than that in the control (*p* < 0.05). The entire body length of nematode in fosthiazate recorded as 371.77 µm, while nematode in distilled water was 501.04 µm. The length of esophageal glands to head was 155.11 µm (fosthiazate) and 199.48 µm (control) respectively. By contrast, the hyaline region was lengthened and turbid compared with the control. Hyaline region length in control and fosthiazate was 27.99 and 57.98 μm, respectively (Table 3).

**Table 3:** Lengths of *H. glycines* J2 at 60 h after replaced fosthiazate with distilled water

	Length (µm)			
Treatment	Stylet	Esophageal glands to head	Hyaline region	Entire body
Fosthiazate	24.16 ± 0.41 b	155.11 ± 3.74 b	57.98 ± 1.13 a	371.77 ± 10.97 b
Water	25.77 ± 0.33 a	199.48 ± 2.03 a	27.99 ± 0.59 b	501.04 ± 8.44 a

**Notes:** Numbers followed “±” referred to standard error. Means within the same column followed by different letters are significantly different (*p* < 0.05) according to LSD test.

### Effect of fosthiazate on hatching of fresh eggs

**Figure 3:**
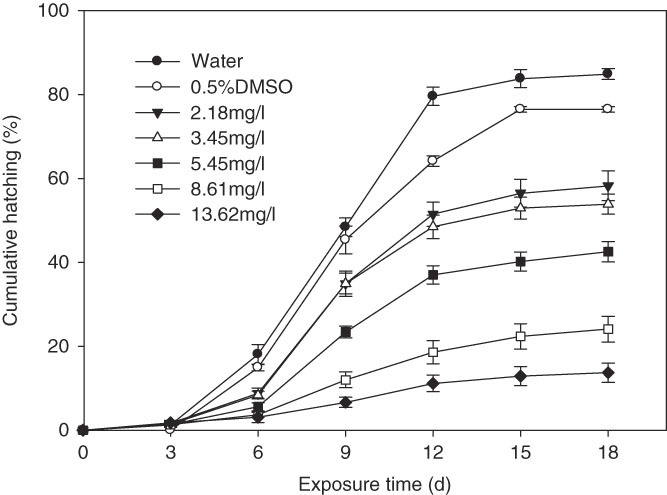
Cumulative hatching percentage of *H. glycines* free eggs exposure to fosthiazate with exposure time. The error bars in the picture referred to standard error.

Free eggs of *H. glycines* exhibited high sensitivity to fosthiazate. The cumulative hatching rate increased with time. The eggs hatched slowly within 0–3 days, and no significant difference was observed among the different treatments (*p* > 0.05) (Fig. 3). Subsequently, significant differences among all concentrations were noted with increasing exposure time (*p* < 0.05). The hatching rate increased at 3 days after incubation and hatched rapidly within 6–12 days. However, the average hatching rate of free eggs in different concentrations of fosthiazate was significantly lower than that of the control in distilled water (*p* < 0.05). Daily hatching rates reached 7.12, 6.69, 5.24, 2.48, and 1.35% in 2.18, 3.44, 5.45, 8.61, and 13.62 mg/l fosthiazate treatments, respectively. Significant difference was observed in cumulative hatching rate after 12-day exposure to different concentrations of fosthiazate (*p* < 0. 05). The cumulative hatching rate of eggs in 2.18, 3.44, 5.45, 8.61, and 13.62 mg/l treatments totaled 58.24, 53.88, 42.54, 24.11, and 13.69% at 18 days after incubation, respectively, and decreased by 26.6, 30.96, 42.3, 60.73, and 71.15% compared with the control treated with distilled water.

### Pot test

Fosthiazate can reduce the number of SCNs. Application of 2 ml fosthiazate at 5.45, 13.62, 34.04, and 85.10 mg/l concentrations significantly affected SCN reproduction in this test. The number of cysts decreased by 43.64, 53.00, 67.21, and 97.94% at 31 days after application, respectively. Application of 0.0272 mg fosthiazate (available ingredient) per pot can reduce the population by 53.0% (Table 4).

**Table 4:** Effect of fosthiazate on control of *H. glycines* in the pot experiment.

Treatment	Amount of fosthiazate (a.i.) (mg)	Average number of cyst	Population decrease (%)
Water	0	412 a	
0.5% DMSO	0	432 a	
2.18 mg/l	0.0044	389 ab	5.43
5.45 mg/l	0.0109	232 bc	43.64
13.62 mg/l	0.0272	194 c	53.00
34.04 mg/l	0.0681	135 cd	67.21
85.10 mg/l	0.1702	9 d	97.94

**Note:** Means within the same column followed by different letters are significantly different (*p* < 0.05) according to LSD test and referred to standard error.

## Discussion

OP compounds have been widely used in controlling agriculture and sanitary insects for their high-efficiency broad spectrum (Jokanović et al., 2011). Fosthiazate, a non-fumigant nematicide, is effective in controlling a wide range of plant parasitic nematodes, including root-knot nematodes, cyst nematodes, and root lesion nematodes (Kimpinski et al., 1997; Pullen and Fortnum, 1999; Zasada et al., 2010).

The nematicidal activity of fosthiazate is not influenced easily by the chemical and physical characteristics of soil (Koyanagi et al., 1998; Wada and Toyota, 2008). Fosthiazate exerts a moderately long residual effect against J2 of plant parasitic nematodes (*M, incognita* and *H. glycines*) and inhibits their movement and invasion in the soil and roots (Koyanagi et al., 1998). Our results confirmed that at low concentration, 13.62 mg/l fosthiazate solution had significant nematicidal activity for *H. glycines* J2 in vitro, and significant decrease in the number of cysts in pot experiment.

A previous study showed that graphite, graphite oxide nanoplatelets, and graphene quantum dots can significantly inhibit the effects on the body length of nematodes after exposure in a concentration-dependent manner (Li et al., 2017). In the present study, fosthiazate reduced nematode mobility and shorten the body length of J2 probably due to the influence of AChE. As far as we know, it is the first report of fosthiazate effect on the nematode morphology. However, the stylet of nematode exposed in fosthiazate is shorter than that of control, which phenomenon cannot be explained, because the stylet is ossific. Fosthiazate solution also exhibited a pronounced effect on *M. incognita* in vitro and egg hatching-inhibition rate; the mortality rate of J2 significantly increased with increasing concentration and treatment time, whereas the motility and infectivity of J2 were significantly depressed (Zou et al., 2011). Similar results showed in *H. glycines* J2, the mortality of J2 reached 86.35% at 12 hr after nematode exposure to 13.62 mg/l fosthiazate solution, whereas the treated J2s showed no recovery of motility when transferred to water. Fosthiazate featured a strong hatching-inhibition effect on eggs in our study, the average hatching rate of free eggs in different concentrations of fosthiazate was significantly lower compared with the control in distilled water. Furthermore, fosthiazate decreased SCN reproduction in the pot test. Similar results also revealed that fosthiazate can delay and suppress hatching of the potato cyst nematode *G. pallida* in in vitro laboratory tests and a glasshouse pot experiments (Woods et al., 1999).

This study demonstrated that fosthiazate exhibits strong toxicity against SCN, including increasing of J2 mortality, and reducing egg hatching and reproduction rates, thus providing evidence to support the use fosthiazate in further studies against *H. glycines*, particularly those carried out in the field.
